# Endoscopic Submucosal Dissection of Papillary Gastric Adenocarcinoma; Systematic Review

**DOI:** 10.3390/jcm9051465

**Published:** 2020-05-14

**Authors:** Chang Seok Bang, Jae Jun Lee, Gwang Ho Baik

**Affiliations:** 1 Department of Internal Medicine, Hallym University College of Medicine, Chuncheon 24253, Korea; baikgh@hallym.or.kr; 2 Institute for Liver and Digestive Diseases, Hallym University College of Medicine, Chuncheon 24253, Korea; 3 Institute of New Frontier Research, Hallym University College of Medicine, Chuncheon 24253, Korea; iloveu59@hallym.or.kr; 4 Department of Anesthesiology and Pain Medicine, Hallym University College of Medicine, Chuncheon 24253, Korea

**Keywords:** endoscopic resection, early gastric cancer, papillary adenocarcinoma

## Abstract

This study evaluated the possibility of endoscopic submucosal dissection (ESD) for early gastric cancer with papillary adenocarcinoma (EGC-PAC). PAC, an uncommon pathologic type of stomach cancer, is classified into differentiated-type histology. However, aggressive features, including a high rate of submucosal invasion, lymphovascular invasion (LVI), and lymph node metastasis (LNM), have been reported in studies with surgical specimens. Treatment outcomes of ESD for EGC-PAC have not been precisely demonstrated. Core databases were sought for the following inclusion criteria: studies of endoscopic resection or surgery of EGC-PAC presenting the following therapeutic indicators; en bloc resection, complete resection, curative resection, recurrence, complications associated with procedures, LVI, or LNM that enabled an analysis of ESD possibility. Overall, 15 studies were included for systematic review. Frequent submucosal invasion and high LVI were noted in EGC-PAC. However, PAC was not significantly associated with LNM. Pooled en bloc resection, complete resection, and curative resection rates were 89.7% (95% confidence interval: 55.3%–98.4%), 85.3% (67.7%–94.2%), and 67% (43%–84.5%), respectively. No LNM was observed if EGC-PAC satisfied the curative resection criteria. ESD seems technically feasible, although a high LVI rate results in a lower rate of curative resection.

## 1. Introduction

Papillary adenocarcinoma (PAC), an uncommon pathologic type of stomach cancer, is characterized by elongated finger-like epithelial projections with a central fibromuscular core [1]. In addition, micropapillary and mixed patterns with tubular components are found in some lesions [2]. The clinical implication or prognostic significance of early gastric cancer with PAC (EGC-PAC) in the stomach has not been clearly established because of its rare entity [3].

Endoscopic submucosal dissection (ESD) is primarily performed in the treatment of EGC with a minimal risk of lymph node metastasis (LNM) [4]. The advantage of ESD over surgery is that its minimal invasiveness enables stomach preservation and acquisition of resected specimen in an en bloc manner. ESD is now considered the standard therapy if the pathologic evaluation of the resected specimen fulfills the absolute criteria (intramucosal EGC with differentiated-type histology (EGC-DH) < 2 cm in the absence of ulceration without lymphovascular invasion (LVI)) [4]. The survival rate after ESD for EGCs that met the absolute criteria was comparable to that achieved with surgery [5]. The indications for ESD have been expanded, and they include mucosal EGC-DH without ulceration irrespective of size; mucosal EGC-DH with ulceration < 3 cm; mucosal EGC with undifferentiated-type histology (EGC-UH) < 2 cm without ulceration; EGC-DH with minute submucosal invasion (≤500 µm of SM1) < 3 cm; and without LVI [6]. Patients with resected specimens satisfying the criteria of curative resection (meeting the absolute or expanded criteria) demonstrate a favorable long-term outcome [7].

The indication for ESD is determined by a combination of histology, size, and morphology of the lesions as described above. These indications are pathologically classified as EGC-DH and EGC-UH [8]. Currently, the categorization of EGC-PAC, based on the Lauren classification and Japanese classification, includes the intestinal-type and EGC-DH, respectively [6,9,10]. EGC-DH is generally considered as having a more favorable prognosis than EGC-UH [11]. Although EGC-PAC is included in the EGC-DH, severe characteristics including a high rate of submucosal invasion, LVI, and LNM, have been suggested in studies with surgical specimens, and treatment results of ESD for EGC-PACs have not been precisely demonstrated [2,3]. In addition, no separate ESD criteria for EGC-PAC have been developed. Therefore, we aimed to assess the possibility of ESD for EGC-PAC.

## 2. Methods

The protocol of this study was registered in the International Prospective Register of Systematic Reviews (PROSPERO) in 2018 (CRD42018115575). The institutional review board approval was waived (collecting data from published studies) [12].

### 2.1. Publication Searching Formula for Systematic Review

MEDLINE-PubMed, Embase, and the Cochrane Library were searched with the common search formula by 2 authors (C.S.B. and J.J.L.). The title and abstracts of all found publications were reviewed and irrelevant publications were excluded. Authors conducted full-text reviews and determined whether the inclusion criteria were met in the remaining studies. The references in the indentified studies were also checked to identify additional related articles. The detailed searching formula is demonstrated in Table S1.

### 2.2. Criteria for Selection of the Literatures

Authors included publications that met the following criteria: 1. Studies with EGC-PAC; 2. Treatment by Endoscopic resection or surgery; 3. Presentation of treatment indicators including en bloc resection, complete resection, curative resection, recurrence, complications associated with procedures, LVI, or LNM that enabled an analysis of ESD possibility; and 5. Full-text articles. Studies that met all the inclusion criteria were searched and selected. The exclusion criteria were as follows: 1. Narrative review; 2. Case; 3. Publication with incomplete data; 4. Meta-analysis; 5. Comments/protocol. Publications satisfying at least 1 of the exclusion criteria were excluded from the systematic review.

### 2.3. Assessment of Quality in Methodology

The assessment of quality in methodology of the included articles used the Risk Of Bias In Non-randomized Studies of Interventions; (ROBINS-I tool) [13]. This tool contains 7 domains and each was rated to exhibit a low, moderate, serious, or critical risk of bias. Overall risk of bias judgement was determined based on the interpretation of each domain level [12,13].

Two authors (C.S.B. and J.J.L.) evaluated the quality in methodology, and any disagreements between the authors were resolved by discussion or consultation with a third author (G.H.B.).

### 2.4. Primary Outcome

Two authors (C.S.B. and J.J.L.) independently collected the primary outcomes and modifiers in each study. The primary therapeutic outcome was as follows: en bloc resection, complete resection, curative resection, recurrence, adverse event of ESD, and LVI or LNM.

Authors previously reported treatment outcomes of ESD for EGC-UH or EGC with mixed-type histology using meta-analysis [8,14]. The same methods previously described were adopted in this study. Narrative synthesis was also conducted with data retrieved from a systematic review.

### 2.5. Statistics

Meta-analysis was conducted using Comprehensive Meta-Analysis Software (version 3, Stat Inc., Englewood, NJ, USA). Heterogeneity was assessed using the I^2^ test, which calculates the % of total variation across the included articles; I^2^ (%) = 100 × (Q − df)/Q, where Q is Cochrane’s heterogeneity statistic, and df signifies the degrees of freedom [15]. Negative values for I^2^ were set to 0, and an I^2^ > 50% was defined as having substantial heterogeneity [16]. Pooled values with 95% confidence intervals (CIs) were measured with a DerSimonian and Laird random-effect model [17]. Publication bias was not assessed because the number of included articles in the meta-analysis was not sufficient. 

## 3. Results

### 3.1. Identified Studies

**Figure 1:**
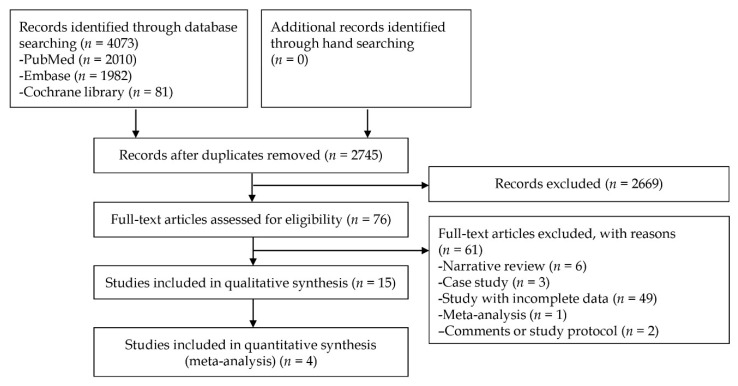
Flow-chart for selection of relevant studies.

Figure 1 is a schematic flow illustrating how the included studies were selected. From the initial search, 4073 research publications were identified by searching core databases; in addition, a manual search was conducted. Overall, 1328 studies were excluded as they were duplicate articles and 2669 publications were additionally excluded in the course of the screening by reviewing titles and abstracts. After a careful review of the full texts of the remaining 76 studies, 61 were excluded from the final evaluation. The reasons for the exclusion during the final evaluation were as follows: narrative review (*n* = 6), case report (*n* = 3), incomplete data (*n* = 49), meta-analysis (*n* =1), and comments, or study protocol (*n* = 2). The remaining 15 studies [10,18,19,20,21,22,23,24,25,26,27,28,29,30,31] were selected in the narrative synthesis; four studies [18,19,20,21] showing common effect-sizes were similarly selected in the meta-analysis.

### 3.2. Quality in Methodology

Most of the articles included showed low or moderate risk for the bias of quality in methodology. Retrospective studies, especially with a single-center data inevitably have a risk of selection bias. Therefore, bias in the “selection of participants” domain might be frequent in this systematic review. However, all the included studies tried to mitigate this bias by including all cases of endoscopic or surgical resections [8].

With regard to the bias in “selection of participants” in this systematic review, most studies showed a low risk. However, publications by Sekiguchi et al. (2013) [24] and Sekiguchi et al. (2015) [26] was rated moderate in terms of risk of bias. Although EGC-PAC is a rare pathologic variant of stomach cancers, different prevalence has been reported according to the study setting. In the analysis of included studies with surgical specimens, a prevalence of approximately 1.9%–15.3% was reported [22,28,31]. Similarly, in the analysis of included studies with endoscopic specimens, a prevalence of approximately 1.4%–6.1% was reported [24,25]. An included study with a combination of endoscopic and surgical specimens showed a prevalence of 18% [27]. However, in a study by Sekiguchi et al. (2013) [24], which included a consecutive series of 1229 endoscopically resected EGCs, the PAC component was of primary concern and its prevalence was 6.1% (75/1229). In addition, in a study by Sekiguchi et al. (2015) [26], which also included a consecutive series of 628 surgically resected EGCs, the PAC component was of primary concern and its prevalence was 62.1% (390/628). Considering that EGC-PAC is usually defined as a tumor in which more than half of the tumor area contains papillary structures which comprises epithelial projections with a central fibrovascular core as a scaffold [18], these should therefore be interpreted differently from other included studies. Therefore, these studies were rated moderate with regard to the risk of bias in the “selection of participants” domain. In addition, a publication by Park [30] included 123 patients who underwent gastrectomy with lymph node dissection due to the presence of LVI after ESD of EGCs; the PAC component was of primary concern and its prevalence was 19% (22/116). Therefore, this study was also rated moderate with regard to the risk of bias in the “selection of participants” domain.

In terms of bias in the “measurement of outcomes”, a study by Huang et al. [27] showed that PAC was a significant risk factor for “worse survival” compared to tubular adenocarcinoma (odds ratio: 3.73, 95% confidence interval [CI]: 1.05−13.23, *p* = 0.042). However, the definition of worse survival was unclear and the overall survival of all included EGCs was only 46.5 ± 26.6 (median follow-up duration: median 51 (range: 11–107) months (11.2% lost to follow-up)). Therefore, this study was rated moderate with regard to the risk of bias in “measurement of outcomes” domain.

Bias in the “selection of reported results” was the most challenging domain in the current systematic review; this aspect is evidenced by the empty spaces in Table 1 and Table S2. As the overall judgement was not different throughout the enrolled publications, subgroup analysis with the quality in methodology was not conducted [8]. A detailed quality in methodology of each study is presented in Table S3.

### 3.3. Clinical Features of the Articles in the Systematic Review

In the 15 retrospective articles [10,18,19,20,21,22,23,24,25,26,27,28,29,30,31] (for the systematic review), a total of 618 EGCs-PAC, and 487 EGCs with PAC component were identified. Of these, four studies [18,19,20,21] commonly reported therapeutic outcomes of ESD; these studies, including 157 endoscopically resected EGCs-PAC were included in the meta-analysis. The published year ranged from 2000 to 2019, and all researches were conducted in Asia (six studies in Korea [18,20,21,29,30,31], five in Japan [22,23,24,25,26], and three in China [10,27,28]), excluding one, which was conducted in Poland [19].

The mean age ± standard deviation of the enrolled patients ranged from 63.4 ± 8.9 to 72.5 ± 9.9 years. Most of the participants in the included studies were predominantly male. In most of the studies, the lesions were predominantly located in the mid- to lower-gastric area. However, a study by Yu et al. [10] reported proximal area predominance. Concerning the morphology of EGC-PAC, most of the included studies showed elevated lesion predominance. However, a study by Min et al. [31] showed flat or depressed lesion predominance. Overall, no robustness was observed in the location or morphology of EGC-PAC. The clinical features of the included articles are demonstrated in Table 1.

### 3.4. Histologic Features of Articles Included in the Systematic Review

Studies [18,19,20,21] with ESD specimens commonly reported smaller sizes of lesions than studies with surgical specimens [10,22,30,31]. Median or mean size of the included ESD and surgical specimens ranged from 21 to 22 mm and 30 to 52 mm, respectively.

For the invasion depth of the lesions, a high rate of submucosal invasion was reported in a majority of the studies, irrespective of the ESD or surgical specimen. The rate of submucosal invasion was reported to range between 15.2% and 41.7% by studies [18,19,20,21] using ESD specimens. Conversely, studies using surgical specimens [10,22,30,31] reported a range of 13.8%–71.4% as the rate of submucosal invasion.

For the LVI of the lesions, two studies with ESD specimens [18,20] reported a high rate of LVI, ranging from 20.8% to 25.3%. However, the study by Park et al. [21] with ESD specimens reported an LVI rate of 3%. In addition, studies with surgical specimens [10,22,30,31] reported a high rate of LVI ranging from 16.9% to 29%. One study with endoscopically resected specimens [24] and two studies with surgical specimens [25,26] commonly reported that PAC component was a significant risk factor for LVI in a multivariate analysis (the odds ratio ranged from 3.1 to 11). Min et al. [31] reported no difference in the LVI rate between tubular adenocarcinoma and PAC in a univariate analysis. However, the absolute value of LVI rate of EGC-PAC in this study was 26.2%, which is included in the high value compared to that of included articles.

The perineural invasion rate is one of the treatment indicators of ESD and only three articles with surgical specimens reported various values ranging from 0% to 7.1% [10,30,31].

Among the studies with ESD specimens, only one research presented on the rate of LNM (4.2%) [18]. Studies with surgical specimens commonly reported higher rates of LNM than those of ESD specimens, ranging from 5.7% to 54% [10,18,21,22,28,30]. However, EGC-PAC was not a significant risk factor for LNM in two studies conducted with surgical specimens [23,28], and lesions that satisfied the ESD criteria showed no LNM in a study with surgical specimens [31]. Only one study with surgical specimens [30] showed that the PAC component was a significant risk factor for LNM in a multivariate analysis presenting a very wide range of CI (OR: 552.5, 95% CI: 1.2-254871.81). The detailed histologic features of the included articles are demonstrated in Table S2.

### 3.5. Treatment Indicators of ESD and Surgery in EGCs-PAC

**Figure 2:**
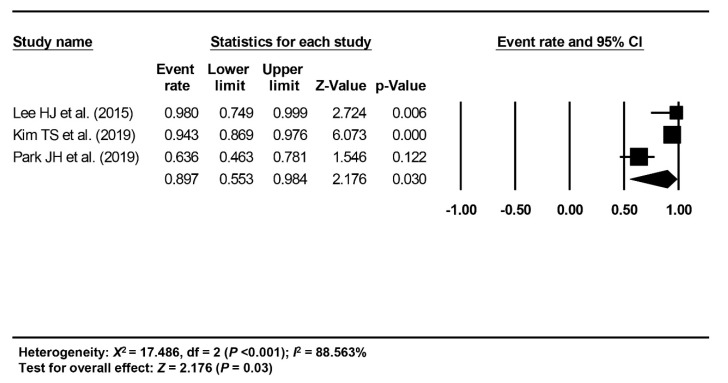
Pooled rate of en bloc resection.

**Figure 3:**
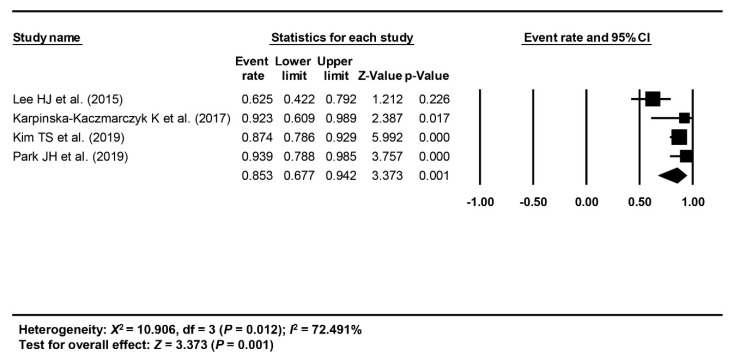
Pooled rate of complete resection.

**Figure 4:**
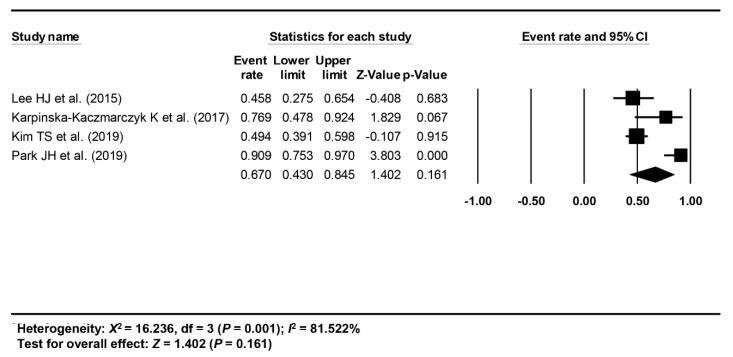
Pooled rate of curative resection.

The short-term treatment outcome of ESD of EGC-PAC was evaluated with the rate of en bloc resection, complete resection, curative resection, post-ESD perforation or bleeding, recurrence, and survival; of these, meta-analysis was possible for the rate of en bloc resection, complete resection, and curative resection. The pooled rates of en bloc, complete, and curative resection were calculated as 89.7% (95% CI: 55.3–98.4%, I^2^ = 88.563%) from three studies [18,20,21] (Figure 2), 85.3% (95% CI: 67.7–94.2%, I^2^ = 72.491%) from 4 studies [18,19,20,21] (Figure 3), and 67% (95% CI: 43–84.5%, I^2^ = 81.522%) from 4 studies [18,19,20,21] (Figure 4), respectively.

The safety of ESD for EGC-PAC was assessed with complications associated with procedures classified as post-ESD bleeding and perforation. Only two studies [20,21] presented the rate of post-ESD bleeding and perforation in the ranges of 5.7%–9.1% and 0%–3.4%, respectively, which were similar to the values reported by studies with ESD for tubular adenocarcinomas.

For the recurrence rate of lesions, two studies with ESD specimens [20,21] commonly reported 0% recurrence during the follow-up. In addition, a study with ESD specimens by Kim et al. [20] reported no local nor extra-gastric recurrences in patients who fulfilled the criteria for curative resection, and the overall metachronous recurrence was 2/38 (5.2%) during the median follow-up of 58 months. However, a study of surgical specimens by Yasuda et al. [22] revealed that 21/65 (32%) patients died of recurrence, and liver metastasis was associated with these cases (13/21 (62%)).

The long-term treatment outcome of ESD for EGC-PAC was assessed using the survival rate. Study with ESD specimens by Park JH et al. [21] reported no mortality during the 39.5 ± 13.2 months follow-up period. In addition, a study with ESD specimens by Kim TS et al. [20] reported no cancer-related deaths in patients with lesions that satisfied the criteria of curative resection. For studies with surgical specimens, it was observed that a 5-year survival rate was not ideal enough, evidenced by values ranging from 63% to 80.5% in two studies [10,22]; however, these rates were commonly not significantly different from those of non-PAC in these studies, as mentioned earlier.

Study by Huang Q et al. [27] only demonstrated that PAC was a significant risk factor for worse survival compared to tubular adenocarcinoma (odds ratio: 3.73, 95% CI: 1.05–13.23, *p* = 0.042). However, the definition of worse survival was unclear, and the overall survival of all included EGCs was only 46.5 ± 26.6, as described earlier.

In detail, the treatment indicators of ESD for EGC-PAC are shown in Table S2.

## 4. Discussion

EGC-PAC is categorized as EGC-DH by the Japanese Classification [6,10]. Therefore, similar ESD indication has been recommended for EGC-PAC and well- or moderately differentiated tubular adenocarcinomas. However, aggressive features of EGC-PAC, such as a high rate of LVI, submucosal invasion, or LNM were frequently indicated by surgical series [2,3]. Considering the fact that studies with surgical specimens probably include a larger size and deeper invasion depth compared to studies with ESD specimens, outcomes suggesting invasiveness could therefore be exaggerated. Notably, treatment outcomes of ESD for EGC-PAC have not been precisely evaluated.

In addition, the current study revealed the discrepancy between studies with surgical specimens and those with ESD specimens. The representative finding was that the median or mean size of the included surgical specimens was larger than that of ESD specimens. However, this aspect was not led to the depth of invasion. A high rate of submucosal invasion was reported by a majority of the included studies; this is regardless of being conducted on ESD or surgical specimens (15.2%–41.7% with ESD specimens vs. 13.8%–71.4% with surgical specimens). Furthermore, a high LVI rate was commonly observed in the enrolled studies, irrespective of the ESD or surgical specimens (20.8%–25.3% with ESD specimens vs. 16.9%–29% with surgical specimens).

Despite the invasive features of EGC-PAC, the analysis revealed that short-term outcomes of ESD met our expectations. Especially, the total rate of complete resection was estimated at 85.3%—a value reasonable enough to justify the performance of ESD even in conditions of a high submucosal invasion rate. Considering that the ESD candidates are highly selected patients after excluding multiple risk factors of non-curative resection, the high rate of complete resection suggests the technical feasibility of performing ESD in EGC-PAC. However, curative resection was not satisfactory, evidenced by an estimated total curative resection rate of 67% in the analysis. Normally, LVI is unpredictable before performing ESD, and high LVI in EGC-PAC could contribute to the overall low rate of curative resection.

Another finding of importance was that no association was observed between the high rate of submucosal invasion or LVI and high LNM in EGC-PAC. Submucosal invasion and LVI are well-known independent risk factors for LNM and for critical prognostic factors in patients with EGC [32,33]. A high rate of LNM was demonstrated by studies with surgical specimens; however, multivariate analysis revealed no significant association between EGC-PAC and LNM. In addition, lesions that satisfied the curative resection criteria demonstrated no LNM in a study with surgical specimens [31]. One plausible explanation is that an association probably existed between LNM and lymphatic invasion grade, stratified by the number of lymphatic tumor emboli in the pathologic specimens, rather than the LVI itself [30,31]. However, this was not measured in any of the enrolled studies.

In terms of recurrence, extra-gastric recurrence rate has been generally reported below 1% after curative resection of EGCs [34,35]. Similarly, studies with ESD specimens of EGC-PAC commonly showed no local nor extra-gastric recurrences in patients who fulfilled criteria for curative resection in the analysis. Only a study with surgical specimens by Yasuda et al. [22] reported a high mortality rate of 32% due to recurrence. However, the size of the specimen included was more than 50 mm (mean value). Moreover, reported indicators of the included lesions were 13.8%, 54%, and 5% of serosal invasion, LNM and peritoneal dissemination, respectively, which suggests the inclusion of highly invasive lesions in this study. However, the methodological quality was not rated highly with regard to the risk of bias in the “selection of participants” domain; overall, this research should be distinctly interpreted.

Long-term outcomes are essential for determining the appropriateness of current ESD indications [20]. Regarding the survival of patients with EGC, a recent Japanese nationwide registry analysis revealed that approximately 100% survival was obtained in the long-term outcomes, including 5-year overall and disease-specific survival rates, if curative resection was achieved, regardless of absolute or expanded ESD indication [36]. Although the overall rate of curative resection was relatively low for EGC-PAC in the analysis, the patients whose lesions were curatively resected showed no cancer-related deaths in studies with ESD specimens [20,21]. In addition, studies with surgical specimens from patients revealed no significant difference in survival between EGC-PAC and non-PAC [10,22].

This is the first study to systematically review and conduct a meta-analysis on the treatment outcomes of ESD for EGC-PAC. One of the strengths of the current study is the strict searching of the articles using a pre-established searching formula. Although there were strengths, several limitations were noted. Firstly, only narrative synthesis was possible in most of the features and treatment outcomes because of the heterogeneity in the study population, methodology, and reported outcomes. Furthermore, quantitative synthesis was only possible in three short-term treatment indicators of ESD. Secondly, all the studies included were conducted in a single-center retrospective analysis setting, which limited the generalization of the study finding. Thirdly, data were not sufficient enough for the evaluation of the indications and the safety indicators of ESD for EGC-PAC.

In conclusion, ESD for EGC-PAC seems a technically feasible modality because of a sufficient en bloc and complete resection rate. However, considering the low rate of curative resection due to the high LVI rate, caution should be exercised in adopting precise ESD indications.

## Figures and Tables

**Table 1:** Clinical features of the articles in the systematic review.

Included Article	Format/Nationality	Number of Patients	Age (Years, Mean ± SD)	Gender	Location of Lesion in Stomach	Type of Endoscopic Morphology
Lee, H.J. et al. (2015) [18]	Retrospective, single-center/Korea	24 patients with EGC-PAC who underwent ESD.	Median 68 (range: 56–80)	19/5	4 (upper third)/20 (lower third)	23 elevated, 1 flat (5 with ulcerations)
Karpińska-Kaczmarczyk, K. et al. (2017) [19]	Retrospective, single-center/Poland	13 patients with EGC-PAC who underwent ESD.	72.5 ± 9.9	8/5	6 (upper third)/7 (middle, lower third)	10 elevated, 3 flat or depressed (1 with ulceration)
Kim, T.S. et al. (2019) [20]	Retrospective, single-center/Korea	87 patients with EGC-PAC who underwent ESD.	65.4 ± 10.1, Median 66 (range: 37–83)	65/21	4 (fundus, cardia)/23 (body)/60 (antrum, angle)	69 elevated, 18 flat or depressed (0 with ulcerations)
Park, J.H. et al. (2019) [21]	Retrospective, single-center/Korea	33 patients with EGC-PAC who underwent ESD.	>65 years: 22/33 (66.7%), ≤65 years: 11/33 (33.3%)	19/14	5 (upper third)/12 (middle third)/16 (lower third)	14 elevated, 10 flat, and 9 depressed
Yasuda, K. et al. (2000) [22]	Retrospective, single-center/Japan	65 patients with EGC-PAC who underwent gastrectomy. EGC-PAC was 10.3% (65/631) of all surgically resected EGCs.	67.7 ± 10.6	49/16	24 (upper third)/41 (lower two-thirds)	55 localized-/10 infiltrative-type
Mita, T. et al. (2001) [23]	Retrospective, single-center/Japan	Among the 35 patients with node-positive differentiated submucosal cancers, 12 EGC-PAC were included.	-	-	-	-
Sekiguchi, M. et al. (2013) [24]	Retrospective, single-center/Japan	Consecutive series of 1229 endoscopically resected EGCs in 1083 patients. PAC component was 6.1% (75/1229).	-	-	-	-
Yamada, T. et al. (2014) [25]	Retrospective, single-center/Japan	143 EGCs who underwent ESD with 2.0 cm or smaller, a differentiated-type adenocarcinoma, and without ulceration (absolute indication of ESD). EGC-PAC was 1.4%.	-	-	-	-
Sekiguchi, M. et al. (2015) [26]	Retrospective, single-center/Japan	Consecutive series of 628 surgically resected EGCs in 628 patients. PAC component was 62.1% (390/628).	-	-	-	-
Huang, Q. et al. (2015) [27]	Retrospective, single-center/China	Among the 438 EGCs (361 by surgery, 59 by endoscopic resection, and 18 by both), 79 EGC-PAC (18%) were included.	-	-	-	-
Fang, C. et al. (2016) [28]	Retrospective, single-center/China	Among the 379 surgically resected EGCs, 58 EGC-PAC (15.3%) were included.	-	-	-	-
Lee, H.J. et al. (2017) [29]	Retrospective, single-center/Korea	56 patients with EGC-PAC who underwent gastrectomy.	67 (range: 49–80)	39/17	5 (upper third)/8 (middle third)/43 (lower third)	43 elevated, 0 flat, and 13 depressed
Park, J.W. et al. (2017) [30]	Retrospective, single-center/Korea	123 patients who had gastrectomy with LN dissection due to presence of lymphatic invasion after ESD of EGCs. PAC component was 19% (22/116).	-	-	-	-
Yu, H. et al. (2017) [10]	Retrospective, single-center/China	59 patients with EGC-PAC who underwent gastrectomy.	65.9 ± 6.9	43/16	33 (cardia, proximal)/26 (non-cardia, distal)	39 elevated, 4 flat, and 16 depressed
Min, B.H. et al. (2018) [31]	Retrospective, single-center/Korea	130 patients with EGC-PAC who underwent gastrectomy. EGC-PAC was 1.9% (130/6710) of all surgically resected EGCs.	65.6 ± 9.4 (in mucosa-confined lesion), 63.4 ± 8.9 (in lesions with submucosal invasion)	94/36	10 (upper third)/22 (middle third)/98 (lower third)	62 elevated, 68 flat or depressed (3 with ulcerations)

EGC-PAC, early gastric cancer with papillary adenocarcinoma; SD, standard deviation; ESD, endoscopic submucosal dissection.

## Supplementary Materials

The following are available online at https://www.mdpi.com/2077-0383/9/5/1465/s1, Table S1: Publication searching formula, Table S2: Histologic characteristics of the articles included in the systematic review. Table S3: Risk of bias evaluation (ROBINS-I assessment tool).
