# Supplementary material for: Endoscopic Submucosal Dissection of Papillary Gastric Adenocarcinoma; Systematic Review
Source: J Clin Med. 2020 May 14;9(5):1465. doi: 10.3390/jcm9051465 (PMC7290846; doi:10.3390/jcm9051465)
Supplement: Supplementary file 1 [file jcm-09-01465-s001.pdf]

Supplementary Table S1. Publication searching formula.

**Database: PubMed-MEDLINE**
**#1 "early gastric cancer"[tiab] OR "gastric cancer"[tiab] OR "stomach neoplasms"[Mesh]: 109776**<br><br>**#2 "Endoscopic submucosal dissection"[tiab] OR "ESD"[tiab] OR "Endoscopic resection"[tiab] OR "Endoscopic mucosal resection"[Mesh]: 10114**<br><br>**#3 "surgical resection"[tiab] OR "gastrectomy"[Mesh]: 81074**<br><br>**#4 #1 OR #2 OR #3: 181543**<br><br>**#5 "papillary"[tiab] OR "carcinoma, papillary"[Mesh] OR "adenocarcinoma, papillary"[Mesh]: 63352**<br><br>**#6 #4 AND #5: 2010**
**Database: Embase**
**#1 'early gastric cancer':ab,ti,kw OR 'gastric cancer':ab,ti,kw OR 'stomach cancer'/exp: 137335**<br><br>**#2 'Endoscopic submucosal dissection':ab,ti,kw OR 'Endoscopic submucosal dissection'/exp OR 'Endoscopic mucosal resection'/exp: 13633**<br><br>**#3 'surgical resection':ab,ti,kw OR 'gastrectomy'/exp: 133928**<br><br>**#4 #1 OR #2 OR #3: 258777**<br><br>**#5 'papillary':ab,ti,kw OR 'papillary'/exp OR 'papillary carcinoma'/exp: 89247**<br><br>**#6 #4 AND #5: 3201**<br><br>**#7 #6 AND ([article]/lim OR [article in press]/lim OR [review]/lim): 1982**
**Database: Cochrane Library**
**#1 early gastric cancer:ab,ti,kw or gastric cancer:ab,ti,kw: 7058**<br><br>**#2 MeSH descriptor: [stomach neoplasms] explode all trees: 2376**<br><br>**#3 Endoscopic submucosal dissection:ab,ti,kw or ESD:ab,ti,kw or Endoscopic resection:ab,ti,kw: 2744**<br><br>**#4 MeSH descriptor: [endoscopic mucosal resection] explode all trees: 59**<br><br>**#5 surgical resection:ab,ti,kw: 9444**

#6 MeSH descriptor: [gastrectomy] explode all trees: 956

#7 #1 or #2 or #3 or #4 or #5 or #6: 18202

#8 papillary:ab,ti,kw: 1569

#9 MeSH descriptor: [carcinoma, papillary] explode all trees: 133

#10 MeSH descriptor: [adenocarcinoma, papillary] explode all trees: 49

#11 #8 or #9 or #10: 1569

#12 #7 and #11: 81 (review 1, trial 80)

Supplementary Table S2. Histologic characteristics of the articles included in the systematic review.

Included article	Size	Invasion depth	LVI	Perineural invasion	Histology of ESD specimen	LNM
Lee HJ et al. (2015) [18]	Median 22 mm (range: 6–59)	Mucosa: 14/24 (m1, m2, m3: 0, 4, 10), Submucosa: 10/24 (41.7%) (sm1, sm2: 4, 6)	5/24 (20.8%) in ESD specimen	-	Pure PAC: 13/24, Mixed papillary type with differentiated adenocarcinoma: 9/24, Mixed papillary type with undifferentiated adenocarcinoma: 2/24	9/49 (18.3%) in surgical specimen, 1/24 (4.2%) in ESD specimen
Karpińska-Kaczmarczyk K et al. (2017) [19]	≤10 mm: 5/13 (38.5%), > 10 mm: 8/13 (61.5%)	T1a: 8/13, T1b: 5/13 (38.5%), submucosal invasion over 500 um: 3/13 (23.1%)	-	-	-	-
Kim TS et al. (2019) [20]	2.1 ± 1.2 mm, Median 18 (range: 2–52)	Mucosa: 51/87, Submucosa: 36/87 (41.4%) (sm1: 12, sm2 or sm3: 24)	Lymphatic invasion: 22/87 (25.3%), venous invasion: 5/87 (5.7%) in ESD specimen	-	Histologic heterogeneity: 14/87 (16.1%)	-
Park JH et al. (2019) [21]	≤20 mm: 26/33 (78.8%), > 20 mm: 7/33 (21.2%)	Mucosa: 28/33, Submucosa: 5/33 (15.2%)	1/33 (3%) in ESD specimen	-	-	13/52 (25%) in surgical specimen
Yasuda K et al. (2000) [22]	52 ± 30 mm	Serosal invasion: 9/65 (13.8%)	Lymphatic invasion: 19/65 (29%), venous invasion: 5/65 (8%)	-	-	35/65 (54%), peritoneal dissemination: 3/65 (5%), liver metastasis: 9/65 (14%), stage III, IV: 24/65 (34%), curative operation: 56/65 (86%)
Mita T et al. (2001) [23]	-	-	-	-	-	Among the 35 patients with node-positive differentiated submucosal cancers, PAC was not a significant risk factor for LNM.
Sekiguchi M et al. (2013) [24]	-	-	PAC component was a significant risk factor for lymphatic involvement in a multivariate analysis (OR: 8.1, 95% CI: 3.2–20.6)	-	-	-
Yamada T et al. (2014) [25]	-	-	Among 143 EGCs in absolute indication of	-	-	-

			ESD, 16 showed SM invasion or LVI. PAC or moderately differentiated adenocarcinoma was a significant risk factor for LVI or SM invasion in a multivariate analysis (OR: 11, 95% CI: 2.9–42)			
Sekiguchi M et al. (2015) [26]	-	-	PAC component was a significant risk factor for lymphatic involvement in a multivariate analysis (OR: 3.1, 95% CI: 1.6–6.0), but the presence of a PAC was not a significant risk factor for LNM.	-	-	PAC was not a significant risk factor for LNM.
Fang C et al. (2016) [28]	-	-	-	-	-	Among the 58 surgically resected EGCs with PAC, 6/58 (10.3%) showed LNM (+). PAC was not a significant risk factor for LNM.
Lee HJ et al. (2017) [29]	Median 30 mm (range: 9–105)	Mucosa: 16/56 (m1, m2, m3: 0, 1, 15), Submucosa: 40/56 (71.4%) (sm1, sm2: 6, 34)	16/56 (28.6%) in surgical specimen	4/56 (7.1%) in surgical specimen	Pure PAC: 34/56, Mixed papillary type with differentiated adenocarcinoma: 14/56, Mixed papillary type with undifferentiated adenocarcinoma: 8/56	10/56 (17.9%) in surgical specimen
Park JW et al. (2017) [30]	-	-	-	-	-	Among 123 patients who had gastrectomy with LN dissection due to presence of lymphatic invasion after ESD of EGCs, 7 (5.7%) showed LNM. PAC component was a significant risk factor for LNM in a multivariate analysis (OR: 552.5, 95% CI: 1.2–254871.81)

Yu H et al. (2017) [10]	<20 mm: 35/59 (59.3%), ≥ 20 mm: 24/59 (40.7%)	Mucosa: 21/59 (m1, m2, m3: 0, 9, 12), Submucosa: 38/59 (64.4%) (sm1, sm2: 21, 17)	10/59 (16.9%) in surgical specimen	0% in surgical specimen	-	8/59 (13.6%) in surgical specimen
Min BH et al. (2018) [31]	3.0 ± 1.2 (in mucosa-confined lesion), 3.4 ± 1.6 (in lesions with submucosal invasion)	Mucosa: 66/130, Submucosa: 64/130 (49.2%) (sm1: 19, sm2: 25, sm3: 20)	Lymphatic invasion: 34/130 (26.2%), venous invasion: 10/130 (7.7%) in surgical specimen. Lymphatic invasion was not different between WD/MD adenocarcinoma and PAC in a univariate analysis.	3/130 (2.3%) in surgical specimen	-	Among 130 EGCs with PAC, 57 mucosal EGCs and 6 submucosal EGCs met the curative endoscopic resection criteria. None of these tumors showed LNM in the surgical specimen.

EGC, early gastric cancer; PAC, papillary adenocarcinoma; ESD, endoscopic submucosal dissection; LVI, lymphovascular invasion; LNM, lymph node metastasis; OR, odds ratio; CI, confidence interval.

Supplementary Table S3. Risk of bias evaluation (ROBINS-I assessment tool).

Risk of bias pre-intervention and at intervention domains				Risk of bias post-intervention domains				
**Study**	**Bias due to Confounding**	**Bias in selection of participants into the study**	**Bias in classification of interventions**	**Bias due to deviations from intended intervention**	**Bias due to missing data**	**Bias in measurement of outcomes**	**Bias in selection of the reported result**	**Overall Assessment of bias**
Lee HJ et al. (2015) [18]	Low	Low	Low	Low	Low	Low	Low	Low
Karpińska-Kaczmarczyk K et al. (2017) [19]	Low	Low	Low	Low	Low	Low	Moderate	Moderate
Kim TS et al. (2019) [20]	Low	Low	Low	Low	Low	Low	Low	Low
Park JH et al.(2019) [21]	Low	Low	Low	Low	Low	Low	Low	Low
Yasuda K et al. (2000) [22]	Low	Low	Low	Low	Low	Low	Low	Low
Mita T et al. (2001) [23]	Low	Low	Low	Low	Low	Low	Low	Low
Sekiguchi M et al. (2013) [24]	Low	Moderate	Low	Low	Low	Low	Moderate	Moderate
Yamada T et al. (2014) [25]	Low	Low	Low	Low	Low	Low	Moderate	Moderate
Sekiguchi M et al. (2015) [26]	Low	Moderate	Low	Low	Low	Low	Moderate	Moderate

Huang Q et al. (2015) [27]	Low	Low	Low	Low	Low	Moderate	Moderate	Moderate
Fang C et al. (2016) [28]	Low	Low	Low	Low	Low	Low	Low	Low
Lee HJ et al. (2017) [29]	Low	Low	Low	Low	Low	Low	Low	Low
Park JW et al. (2017) [30]	Low	Moderate	Low	Low	Low	Low	Moderate	Moderate
Yu H et al. (2017) [10]	Low	Low	Low	Low	Low	Low	Low	Low
Min BH et al. (2018) [31]	Low	Low	Low	Low	Low	Low	Low	Low
